# Chitosan scaffolds with mesoporous hydroxyapatite and mesoporous bioactive glass

**DOI:** 10.1007/s40204-023-00217-x

**Published:** 2023-02-09

**Authors:** Ana Sofia Pádua, Lígia Figueiredo, Jorge Carvalho Silva, João Paulo Borges

**Affiliations:** 1 I3N/CENIMAT, Materials Science Department, NOVA School of Science and Technology, New University of Lisbon, Lisbon, Portugal; 2 Bioceramed S.A., Rua José Gomes Ferreira 1, Arm D, São Julião Do Tojal, 2660-360 Loures, Portugal; 3 I3N/CENIMAT, Physics Department, NOVA School of Science and Technology, New University of Lisbon, Caparica, Portugal

**Keywords:** Mesoporous hydroxyapatite, Mesoporous bioactive glass, Chitosan, Scaffold, Bone tissue engineering

## Abstract

Bone regeneration is one of the most well-known fields in tissue regeneration. The major focus concerns polymeric/ceramic composite scaffolds. In this work, several composite scaffolds based on chitosan (CH), with low and high molecular weights, and different concentrations of ceramics like mesoporous bioactive glass (MBG), mesoporous hydroxyapatite (MHAp) and both MBG and MHAp (MC) were produced by lyophilization. The purpose is to identify the best combination regarding optimal morphology and properties. The tests of the scaffolds present a highly porous structure with interconnected pores. The compression modulus increases with ceramic concentration in the scaffolds. Furthermore, the 75%MBG (835 ± 160 kPa) and 50%MC (1070 ± 205 kPa) samples are the ones that mostly enhance increases in mechanical properties. The swelling capacity increases with MBG and MC, respectively, to 700% and 900% and decreases to 400% when MHAp concentration increases. All scaffolds are non-cytotoxic at 12.5 mg/mL. The CHL scaffolds improve cell adhesion and proliferation compared to CHH, and the MC scaffold samples, show better results than those produced with just MBG or MHAp. The composite scaffolds of chitosan with MBG and MHAp, have revealed to be the best combination due to their enhanced performance in bone tissue engineering.

## Introduction

Autografts, namely, osteogenic and osteoinductive, are the supreme ways of enhancing bone regeneration in applications as diverse as orthopaedic trauma surgery, correction of congenital bone defects or spinal fusion (Salgado et al. 2004; Giannoudis et al. 2005; Habibovic and Groot 2007; Bhatt and Rozental 2012; de Melo Pereira and Habibovic 2018). Nevertheless, failure rates between 5 and 13% and complications rates (including chronic pain, blood loss, nerve injury, hernia formation, infection, arterial injury) between 8.5% and 20%, have been reported (Kaing et al. 2011; Bhatt and Rozental 2012; Kurien et al. 2013). This has led to research possibility on the use of biomaterials for bone regeneration and the development of alternative bone graft options such as ceramic, polymeric and composite scaffolds (Madihally and Matthew 1999; Rodríguez-Vázquez et al. 2015).

The ceramics hydroxyapatite (HAp) and bioactive glass (BG) are the most used materials to fabricate the bone substitutes available on the market. Alone or in combination with other materials, they present versatility, due to different forms, porosities, pore sizes and structures achievable (Habibovic and Groot 2007; Habibovic et al. 2008; Erol and Boccaccini 2011; García-Gareta et al. 2015). Of the recently developed structures, the mesoporous structure has improved properties regarding both morphology and mechanical response. The morphology showed outstanding surface area values and porosity, conferring high efficiency in chemicals incorporation and subsequent release (in situ drug delivery) of antibiotics, anticancer drugs or cytokines (Qiao et al. 2017; Munir et al. 2018). The improved mechanical properties include higher resistance after swelling and assays of simulated body fluids (SBF) (Arcos et al. 2011).

The mesoporous structure is obtained through the incorporation of surfactants in sol–gel process (Arcos et al. 2011) or a surfactant catalyst in the micro-wave synthesis (Zhou et al. 2018). But, ceramic bone grafting materials still have some flaws such as: low fracture strength, low bending strength, brittleness and degradation rates difficult to predict (Giannoudis et al. 2005; Jones 2005; Karageorgiou and Kaplan 2005; De Lo ng et al., 2007; Dorozhkin 2010, 2013; Erol and Boccaccini 2011; Wagoner Johnson and Herschler 2011; García-Gareta et al. 2015; Wegst et al. 2015).

In order to improve ceramic bone graft properties, such as enhanced mechanical properties with scaffold brittleness reduction and biological performance (Wubneh et al. 2018; Ahmadipour et al. 2022), and satisfy clinical requirements (mass transport, vascularization, and host tissue integration) (Webber et al. 2015), a polymer, such as chitosan (CH), is added to scaffold constitution. CH is composed of β(1 → 4)-linked 2-acetamido-2-deoxy-β-d-glucose (*N*-acetylglucosamine) obtained from the partial deacetylation of chitin (Rodríguez-Vázquez et al. 2015). The degree of deacetylation (DD), crystallinity and molecular weight (MW) are the main aspects in which chitosan can be modified to obtain different physical and mechanical properties (Jain et al. 2013; Rodríguez-Vázquez et al. 2015; João et al. 2017).

Chitosan has a molecular weight in between 50 and 2000 kDa and DD between 40 and 98%. Due to these properties, chitosan has a strong hygroscopic nature, can improve the survival rate of osteoblasts, promote osteoblast differentiation and matrix mineralization (Madihally and Matthew 1999; Jain et al. 2013; João et al. 2017).

The improvement of osteoconduction enhances the bond between bone tissue and the scaffold (Habibovic et al. 2008). In addition, the increase of mechanical strength, pore size, and bioactivity is a result of polymeric and ceramic composite scaffolds (Thein-Han and Misra 2008; Peter et al. 2010a,b). The use of CH and mesoporous ceramics, such as mesoporous Hap (MHAp) or mesoporous BG (MBG), allows easier drug loading and delivery to enhance anti-inflammatory responses, osteointegration, osteoinduction, and, ultimately, a faster bone regeneration (Baino et al. 2017; Cai et al. 2018; Yu et al. 2021). Furthermore, a controlled optimization with very specific macrostructure, microstructure, protein coating and chemical composition can lead to an osteoinductive response (Sikavitsas et al. 2001; Salgado et al. 2004; Jones 2005; Karageorgiou and Kaplan 2005; Dorozhkin 2013).

Nevertheless, there is no study on the effect of adding MHAp or MBG in a composite material for bone regeneration applications. Therefore, the main objective of this work is to produce composite scaffolds of CH with different concentrations of MHAp and MBG by lyophilization and compare them with CH scaffolds, with low and high molecular weights, and composite scaffolds using just mesoporous Hap (MHAp) or mesoporous BG (MBG) to determine the most promising formulation in terms of bone regeneration applications.

## Materials and methods

### Materials

Chitosan with a low molecular weight (CHL) of 100 kDa and a degree of deacetylation (DD) of 80%, and chitosan with a high molecular weight (CHH) of 500 kDa and a 79.4% DD were supplied by Bioceramed (Portugal). Lactic acid (2-hydroxypropanoic acid), purchased from HiMedia (minimum assay = 99.0%), was used to dissolve CH.

In MBG syntheses, following chemicals: tetraethyl orthosilicate (TEOS, Si(OC_2_H_5_)_4_, from Aldrich Chemistry), triethyl phosphate (TEP, PO(C_2_H_5_)_3_, from Fluka Analytical), calcium nitrate tetrahydrate (Ca(NO_3_)_2_·4H_2_O from VWR Chemicals, Pluronic F127 (F127), from Aldrich Life Science and Ethanol from Sigma- Aldrich were used.

In MHap syntheses, the chemicals: calcium nitrate tetrahydrate (Ca(NO_3_)_2_·4H_2_O from VWR Chemicals, phosphoric pentoxide (P_2_O_5_,) from Sigma-Aldrich, F127 from Aldrich Life Science and Ethanol from Sigma-Aldrich) were used.

Ultrapure Water (Milli-Q) was used for the preparation of all solutions and samples.

For the biodegradation test, lysozyme from chicken egg white from Lysozyme BioChemica was used.

Human osteosarcoma cells (SaOS-2 cell line), cultured in McCoy’s 5A (Sigma-Aldrich) medium were used in cytotoxicity and adhesion tests. In both tests, population quantification was a result of resazurin (from Alfa Aesar) reduction by viable cells. In the cytotoxicity tests, the positive control was obtained using dimethyl sulfoxide (DMSO). Helix NP™ Green nuclear stain from BioLegend was used for the cell fluorescence assay.

### Preparation of mesoporous scaffolds

The scaffolds were fabricated by lyophilization of solutions of CH and CH with ceramic mesoporous materials. The ceramic mesoporous materials were produced by sol–gel method using a non-ionic block copolymer F127 at a concentration of 21% of precursor mass, following Yan et al*.* MBG synthesis (Yan et al. 2005) and Fathi et al. for the MHAp synthesis (Fathi and Hanifi 2007).

The polymeric scaffolds were prepared by dissolving 2% (w/v) CH in a 2% (v/v) lactic acid solution and stirring for 2 h. The composite scaffolds had different fractions of MHApor MBG as 25%, 50% and 75% mass ratios of ceramic/CH and in the MC composites (with both MHAp and MBG), the ceramics were always at a 1:1 ratio of 25% and 50% ceramic/CH mass ratios. The ceramics were ultrasonically dispersed (Ultrasonic Processor UP400S from Heilscher) in 2% (v/v) lactic acid until all the clusters were disaggregated and then the CH solution was added while the solution was being stirred. Next, the composite dispersions were vigorously mixed using a magnetic stirrer for 2 h to obtain a homogeneous mixture.

After obtaining the homogenous dispersions, the solutions were poured into Teflon moulds and kept in the freezer overnight, to remove air bubbles and level the solution’s surfaces. Then, the moulds were transferred to the freeze dryer (FreeZone Triad Cascade Benchtop, Labconco, 7400030 model). Lyophilization was performed at 0.1 mbar for 25 h. In order to completely remove the lactate still present inside the scaffolds, these were neutralized in 10% (v/v) NaOH bath, followed by 48 h dialysis (until reaching a pH of around 7) and again lyophilized (VaCo 2 by Zirbus).

### X-ray diffraction (XRD)

The X-ray diffractograms were used to determine the crystal phases of different samples. These analyses were carried out at room temperature using a X’Pert PRO PANAlytical X-ray powder diffractometer (CuK-alpha radiation) operating at a voltage of 45 kV in the range 10° < 2θ < 90° with a 0.033° step size.

### Porosimetry

The porosity of the scaffolds was calculated by Archimedes method, using a Sartorius BP110 S balance. The samples were previously swelled in a PBS bath for 7 days. This analysis used three replicas for each scaffold.

### Scanning electron microscopy (SEM)

The morphology of the composite scaffolds was examined in a field emission SEM (Hitachi S-2700). The samples were frozen and broken in liquid nitrogen, mounted on aluminium platforms for horizontal/transversal view and sputter-coated with a gold–palladium conductive layer (Q3000T D Quorum sputter coater). The images were taken at an accelerating voltage of 15 kV and several magnifications.

### Compression modulus

The mechanical properties of the scaffolds were measured with a testing machine from Rheometric Scientific (Minimat Firmware version 3.1), equipped with a 100 N load cell, at a crosshead speed of 1 mm.min^−1^ at room temperature and in compression mode. The compression modulus of the scaffolds was calculated from the slope of the stress–strain plot at 5% to 10% strain range of ten replicas (Tamplenizza et al. 2015).

### Fourier-transform infrared spectroscopy (FTIR)

Fourier-transform infrared (FTIR) spectroscopy was performed on different materials, using a Thermo Nicolet 6700 spectrometer at Attenuated Total Reflectance (ATR) mode in a wavenumber range of 4000–500 cm^−1^.

### Swelling

The water uptake, or swelling, study was performed in PBS at pH 7.4 at 37 °C using three replicas for each material tested. With the dry weight (W_0_) of the scaffold registered, scaffolds were placed in PBS buffer solution at pH 7.4 for 12 h, 24 h, 48 h, 72 h and 96 h. The excess water in the interior and surface of the sample, was removed with filter paper (Filter-Lab 1300/80) and wet weight (${W}_{\mathrm{f}}$) was recorded for the three replicas. The swelling degree was determined by the following ratio:

$$E=\frac{{W}_{f}-{W}_{0}}{{W}_{0}}\times 100.$$

### Biodegradation

Degradation of the composite scaffold was studied in PBS medium, with ionic force of 0.06 and 5 µg/mL of lysozyme (Davies et al. 1969 and Freier et al. 2005). The samples were immersed in the degradation solution and incubated at 37 °C in closed falcon tube 14 days, with enzyme refreshing in 2-day periods. In the end of each interval, the scaffolds were taken from the degradation medium and rinsed methodically with Milli-Q to remove ions adsorbed on surface.

The biodegradation was quantified by the sample’s variation of weight in the three replicas (after a lyophilization as a drying process) (Sashiwa et al. 1990). The quantification of the remaining weight is given by:

$$\mathrm{Weight\,remaining\,}\left(\mathrm{\%}\right)=100-\frac{{W}_{0}-{W}_{\mathrm{f}}}{{W}_{0}}\times 100.$$

### Bioactivity

For the bioactivity tests, the different samples were cut in squares of 5 mm edge and immersed in 30 mL of SBF solution, reported by Kokubo et al., (Kokubo and Takadama 2006), to guarantee the ratio ${V}_{S}={{S}_{A}}/{10}$, where ${V}_{S}$ is the volume of SBF in mL and ${S}_{A}$ is the sample’s apparent surface area in mm^2^. The samples were incubated at 37 °C in closed falcon tubes for 3, 6, 12, 24, 48, 72 h and 7 days with two replicas of each analysis (Kokubo and Takadama 2006). After the specified periods, to remove non-adsorbed minerals, scaffolds were washed five times with Milli-Q water. Then, in order to identify apatite precipitation, the scaffolds were dried at ambient conditions and viewed using SEM (Kokubo and Takadama 2006; Peter et al. 2010a).

### Cell culture studies

#### Cytotoxicity

The cytotoxicity tests were performed according to ISO 10993–5 standard using the extract method. Samples were sterilized with ethanol and irradiated with UV for 2 h and followed by 2 h remaining still at 80 °C, to guarantee ethanol evaporation. For extract preparation, the scaffolds were immersed in McCoy’s culture medium at a ratio of 25 mg/mL (mass of sample/volume of culture medium). These preparations, as well as some extra medium for the extract dilution and the negative control, were incubated at 37˚C under a controlled 5% CO_2_ atmosphere for 48 h.

The Saos-2 cells were seeded at a concentration of 30 k cells/cm^2^ in the wells and incubated for 24 h. Then, the medium was exchanged for the extract and two dilutions (12.5 mg/mL and 6.25 mg/mL) were made, each with four replicates. For the resazurin test, a negative control (cells cultured in a standard, non-cytotoxic environment) and a positive control (cells in a cytotoxic environment, created through the addition of 10 µL of DMSO, a cytotoxic agent, to normal culture medium) were set.

The extracts and controls were incubated for 48 h and then media were replaced by a 1:1 solution of resazurin (dissolved at a concentration of 0.04 mg/mL in PBS) and McCoy’s medium and incubated for 3 h. The cell activity was evaluated by measuring the absorbance of the medium at 570 nm (absorption maximum of resorufin) and 600 nm (absorption maximum of resazurin) in a microplate reader (Biotek ELx 800UV) (Carmo 2018).

#### Cell adhesion

The ability of the scaffolds to support cell metabolism was evaluated through cell adhesion and proliferation studies. The scaffolds were sterilized in the same way as for the cytotoxicity tests. Then, the materials for the cell culture and material controls were fixed in Teflon supports and placed in a 24-well plate.

The Saos-2 was seeded at a concentration of 30 k cells/cm^2^ directly over the sample’s surface and, for the cell controls, in the wells. The cells were maintained in McCoy’s medium and incubated at 37 °C in a controlled 5% CO_2_ atmosphere for 24 h.

The cell adhesion rate was determined by evaluating the reduction of resazurin to resorufin by metabolically active cells. For this process, the medium was substituted by a 1:1 solution of resazurin/McCoy’s medium and incubated for 4 h. Control wells, containing the resazurin/McCoy’s mix and McCoy’s (both wells without cells) were also incubated. The cell activity was evaluated by measuring the absorbance of the medium at 570 nm and 600 nm in a microplate reader (Biotek ELx 800 UV) (Carmo 2018). The resazurin assay was repeated at 3, 6, 8 and 10 days for evaluation of the cell proliferation for each of the six replicas of all the materials.

After the last readings, the materials were removed from the multi-well plate, washed with PBS and fixed with a 3.7% paraformaldehyde solution, incubated at room temperature for 15 min. Finally, the samples were washed with water and stained with Helix NP™ Green and observed using fluorescence microscopy.

### Statistical treatment

All average values calculated and displayed in the graphics include a representation of the experimental standard deviation with a vertical segment. Statistical analysis was performed using the one-way analysis of variance (ANOVA) with several confidence intervals. The value of *p* < 0.05 was considered to be statistically significant.

## Results

### FTIR

**Fig. 1:**
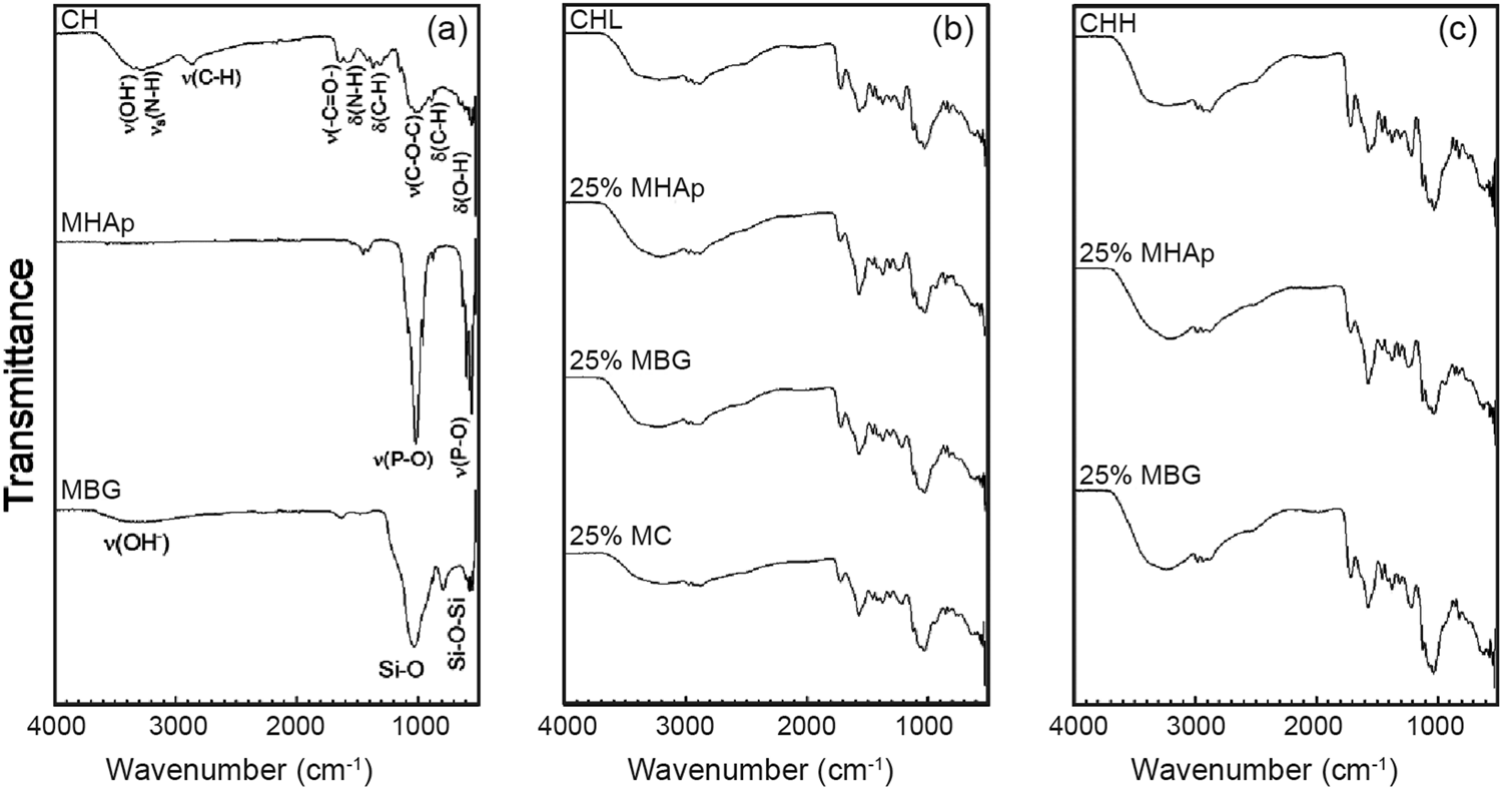
FTIR analysis of the main components of materials (**a**), CHL scaffolds (**b**) and CHH scaffolds (**c**)

The FTIR spectra of MHAp in Fig. 1a shows the inorganic carbon ions (CO_3_^2−^) located at 1456 cm^−1^ and 1411 cm^−1^ and from 742 to 878 cm^−1^, a result of asymmetric bending mode of CO_3_^2−^ (Franco et al. 2012 and João et al. 2016). The main bands of MHAp are present in broad peaks centred at 1115 cm^−1^, 1020 cm^−1^, in the range 925 cm^−1^ to 960 cm^−^1 and at 580 cm^−1^. The first two bands correspond to P-O vibrating bonds of the phosphate groups in the asymmetric stretching mode, the third band corresponds to a symmetric stretching mode of the ion and the last to the asymmetric bending mode of PO_4_^3−^ (Thein-Han and Misra 2008; Franco et al. 2012; Pighinelli and Kucharska 2014; João et al. 2016).

The structural MBG bonds are present in the peak at 1150 cm^−1^, the range from 820 cm^−1^ to 780 cm^−1^ and at 569 cm^−1^. They correspond to the Si–O–Si asymmetric stretching, symmetric stretching or vibration modes and bending mode, respectively. The Si–O bond with the Q^2^ and Q^3^ units can be seen at 1032 cm^−1^ and the Q^1^ and Q^2^ units at 947 cm^−1^ (Arcos et al. 2011 and Stan et al. 2011).

The FTIR spectra of both CHL and CHH show a broad band in the range of 3270 to 3365 cm^−1^ that represents the overlap of N–H (3280 cm^−1^) and O–H (3358 cm^−1^) stretching vibration. The bands around 2867 cm^−1^ and 2921 cm^−1^ correspond to asymmetric and symmetric stretching modes of C–H of CH_2,_ respectively. The symmetric stretching is less intense than the asymmetric stretching, so it is partially hidden with the overlapping of the bands (Molaei et al. 2015; Queiroz et al. 2015; João et al. 2017).

The band around 1645 cm^−1^ shows the C=O stretching of amide I from the residual presence of *N*-acetyl groups. The 1311 cm^−1^ band is due to the N–H bending of amide II (Thein-Han and Misra 2008; Correia et al. 2011; Queiroz et al. 2015). The 1581 cm^−1^ band represents the N–H bending of the primary amine. The absorption signals at 1423 and 1372 cm^−1^ are attributed to all hydrocarbonate bonds, CH_2_ bending and CH_3_ symmetrical deformations (Queiroz et al. 2015).

The stretching of the C–O–C bridge is present in the wavenumber of 1149 cm^−1^ and in the 1065 to 1016 cm^−1^ range, respectively, to an asymmetric stretching and a simultaneous symmetric and asymmetric stretching vibrations of the ester bond (Thein-Han and Misra 2008; Correia et al. 2011; Song et al. 2014).

The CH out-of-plane bending of the ring of monosaccharides is visible by a band at 896 cm^−1^. The band around 650 cm^−1^ represents the bending deformation of O–H on the polymeric structure (Thein-Han and Misra 2008 and Queiroz et al. 2015).

The composite scaffolds in Fig. 1b, c present the bands of all the ceramic and polymeric materials used. It is possible to observe the intensity reduction of a 1000 cm^−1^ band which corresponds to the major MBG and MHAp bands. This variation is due to the overlap of symmetric and asymmetric stretching vibrations of the ester bond with Si–O Q^2^ and Q^3^ units. The addition of MHAp to CH also induces the formation of 560 cm^−1^ peak for the asymmetric bending mode of PO_4_^3−^ in the CH spectra. The MBG composite reduces the peak of Si–O–Si symmetric stretching at 800 cm^−1^, compared to the ceramic spectrum.

### X-Ray diffraction

**Fig. 2:**
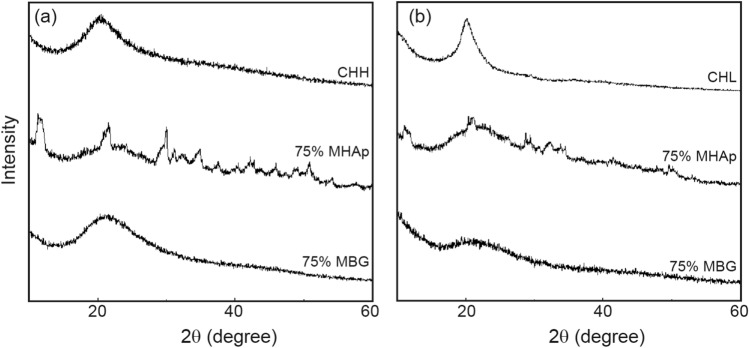
XRD of CHL scaffolds (**a**) and CHH scaffolds (**b**)

The XRD results presented in Fig. 2 show that all the scaffolds produced have a peak approximately at 20°. This peak is attributed to the chitosan present in the sample since this material has a slightly crystalline structure (Jampafuang et al. 2019). The scaffolds with MBG only show the CH peak, though the scaffolds with a high concentration of MHAp display crystalline peaks of the ceramic and the CH peak.

### Porosimetry

All values presented in Table 1 are between 85 and 95% of porosity. The increase of ceramic concentration did not present an evident of direct variation in porosity values. However, addition of ceramic to the matrix tended to reduce the scaffold porosity.

**Table 1:** Porosity of the produced samples

		CHL	CHH
Ceramics	0%	93% ± 2%	95% ± 2%
MHAp	25%	90% ± 1%	89% ± 3%
	50%	85% ± 2%	84% ± 2%
	75%	89% ± 3%	87% ± 2%
MBG	25%	91% ± 2%	91% ± 4%
	50%	90% ± 1%	87% ± 2%
	75%	95% ± 2%	90% ± 1%
MC	25%	94% ± 3%	
	50%	90% ± 1%	

One of the major factors in successful scaffold outcome is high porosity: a network of interconnected large pores without occluded passages that allows for cell migration and proliferation during bone ingrowth, provides open space for nutrient and oxygen supply and further vascularization in newly formed bone tissues (Kang and Chang 2018 and Abbasi et al. 2020).

### Scanning electron microscopy

**Fig. 3:**
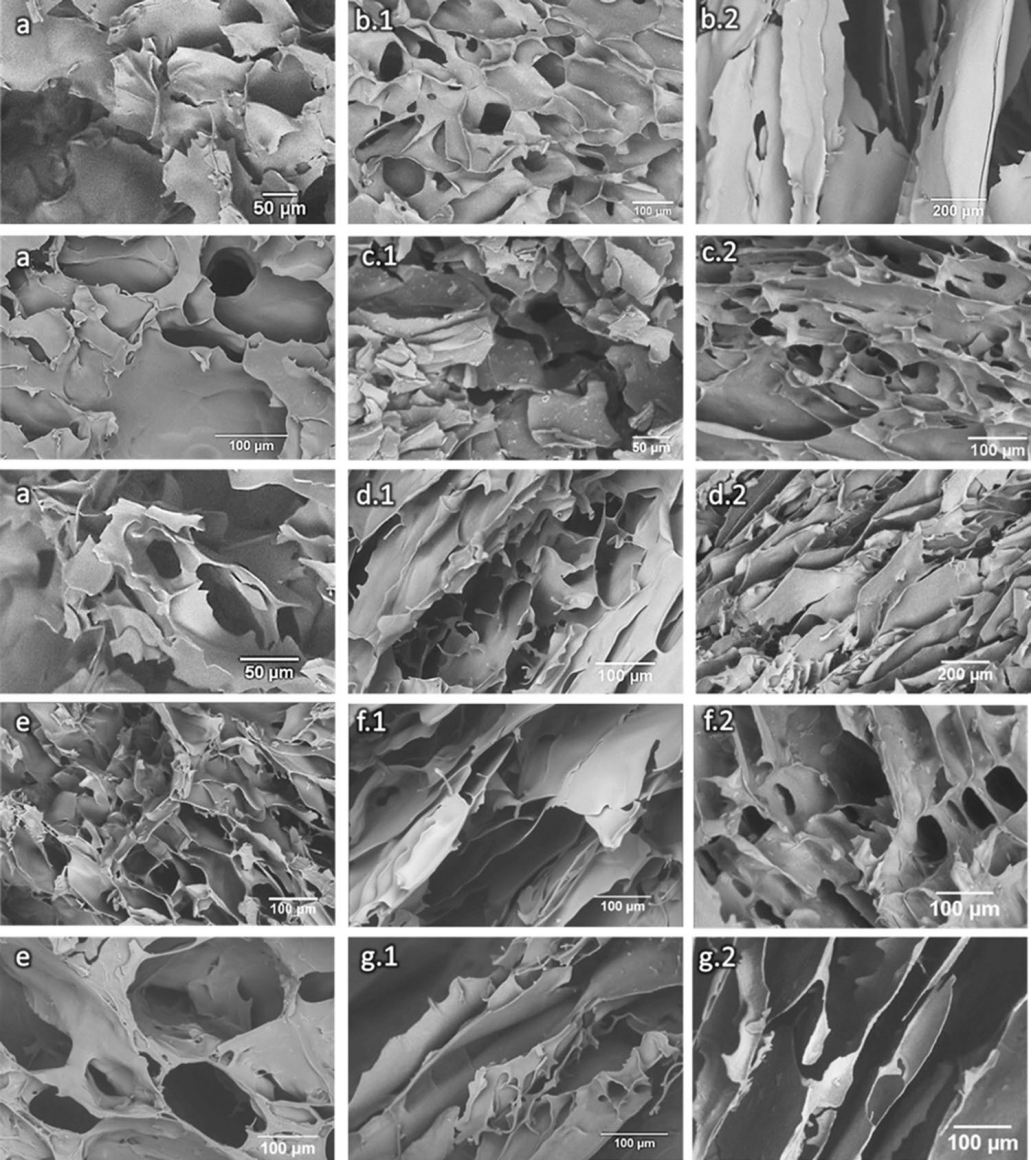
SEM imaging of CHL scaffolds (**a**), CHH scaffolds (**e**) and composite scaffolds with CHL + MHAp (**b**), CHL + MBG (**c**), CHL + MC (**d**),CHH + MHAp (**f**) and CHH + MBG (**g**) at 25%w/w (1) and high (75% for MHAp and MBG, and 50% for MC) (2) ceramic concentrations

The SEM images of all the scaffolds produced are shown in Fig. 3. All scaffolds present an interconnected porous structure and slightly preferential orientation as visible in (b2), (d2), (f1), (g1) and (g2).

### Swelling

**Fig. 4:**
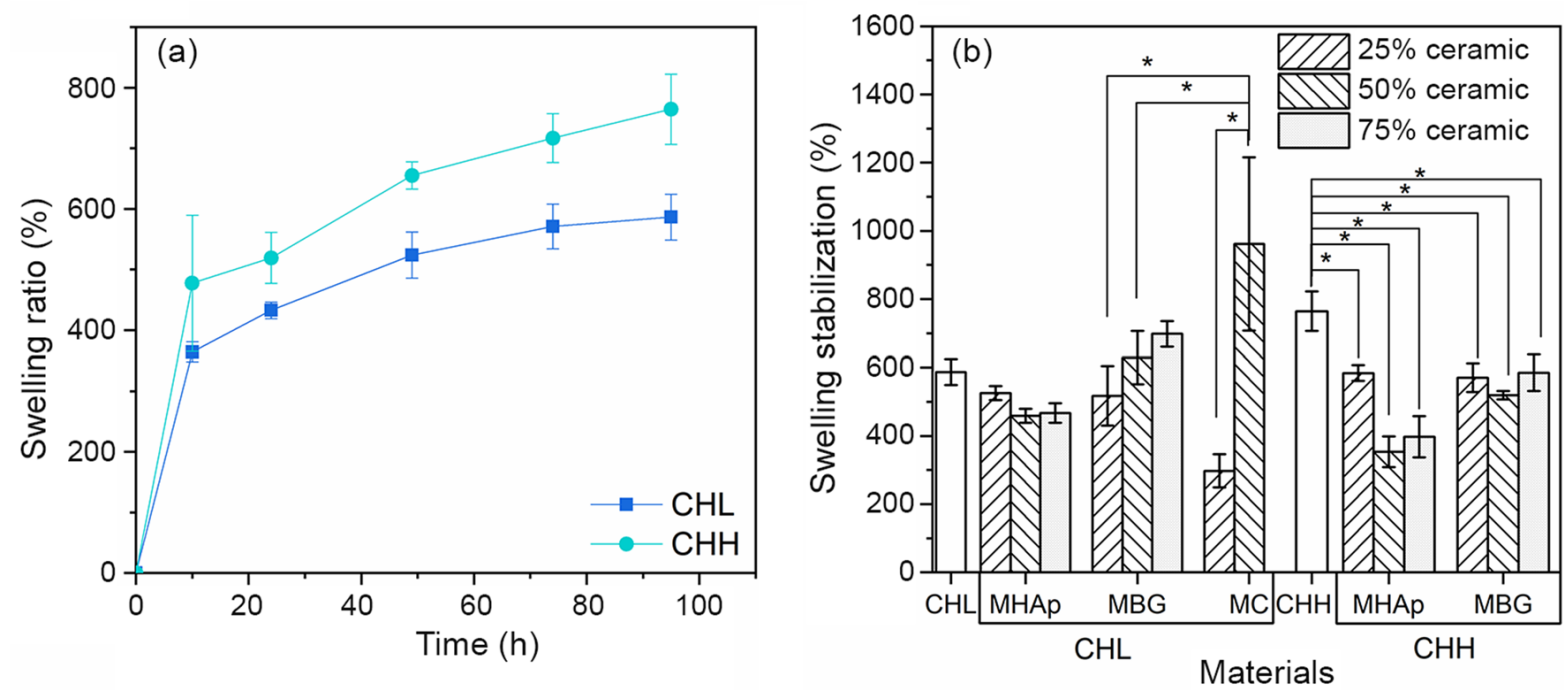
Swelling behaviour of polymeric scaffolds (**a**) and composite scaffolds after 4 days in PBS (**b**). *Significant difference with *p* < 0.05 (*n* = 3)

Figure 4a presents the swelling behaviour of the polymeric scaffolds produced, and the swelling stabilization percentage is shown in Fig. 4b. The comparison between polymeric scaffolds shows that both samples have acquired a plateau, and that CHH has shown a significant (*p* < 0.05) higher swelling capacity than CHL scaffolds.

The CHL + MHAp scaffolds present a slightly decreased swelling capacity when compared to CHL scaffolds but the differences are not statistically significant. The CHL + MBG scaffolds present the opposite response with the increase of swelling capacity with the increase in ceramic content. The MC scaffolds have demonstrated to have a significant difference (*p* < 0.01) between 25 and 50%. While the 25% has presented the lowest swelling capacity of all CHL-based composites, the 50%MC has the highest value. The CHL + 50%MC sample exceeds every other scaffold swelling capacity even the ones with higher ceramic concentration, with the exception of CHL + 75%MBG.

The CHH scaffolds present a more constant behaviour with a significant decrease in swelling capacity in both MHAp and MBG composite scaffolds. Where MHAp significantly decreases swelling capacity with the increase of ceramic content from 25 to 50% (*p* < 0.01), the MBG composites show a constant behaviour for all ceramic concentrations.

### Biodegradability

**Fig. 5:**
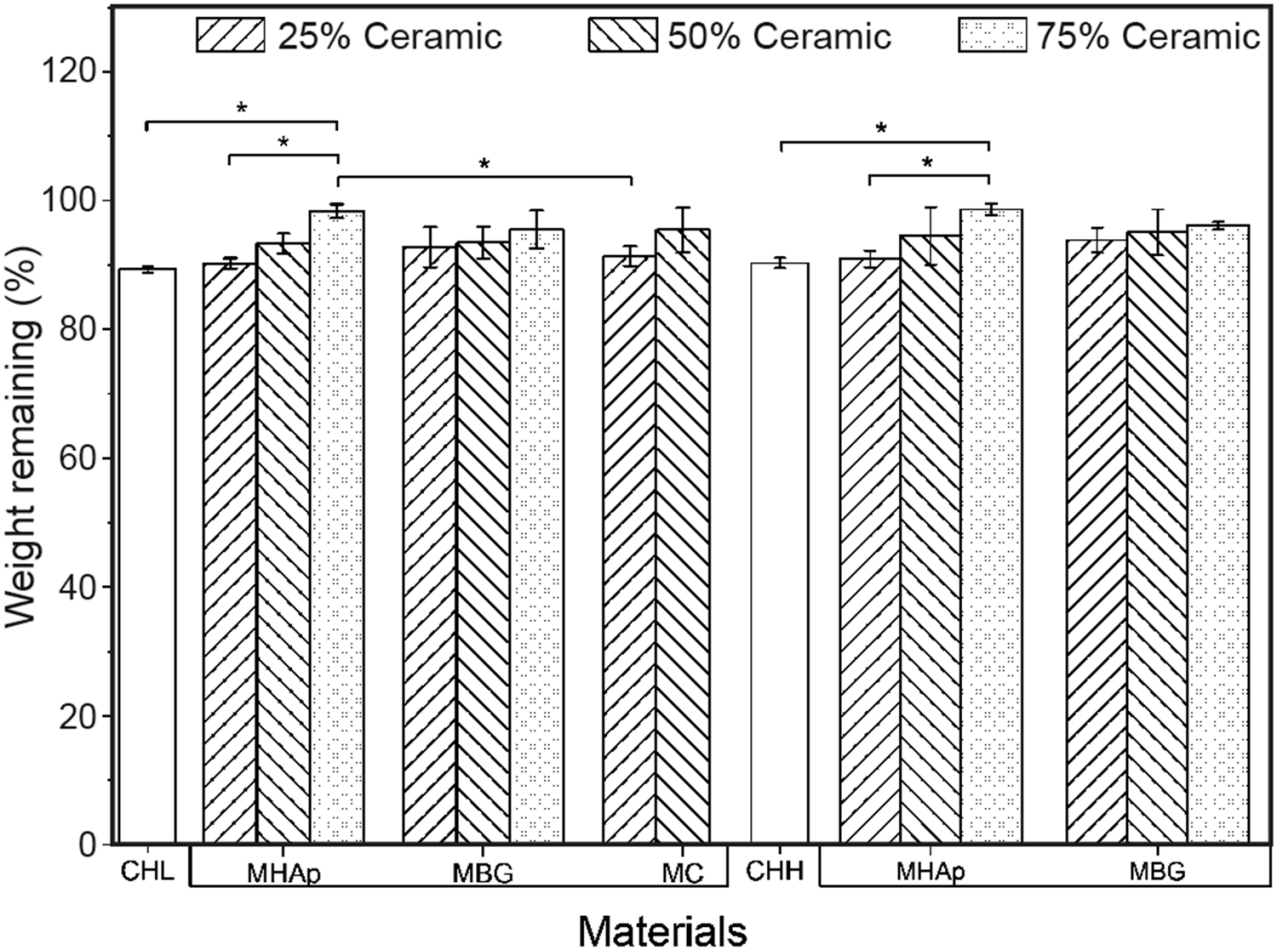
Biodegradation behaviour of scaffolds after 14 days in lysozyme of 5 µg/mL. *Significant difference with *p* < 0.05 (*n* = 3)

The biodegradation behaviour of the material is a crucial factor on the long-term performance of tissue-engineered cell–material construct, as cells need a stable material to adhere and proliferate. (Rodríguez-Vázquez et al. 2015, Lončarević et al. 2017a) In order to analyze the polymeric membranes biodegradation profile, the scaffolds were immerged in PBS containing lysozyme for 14 days. The obtained results are presented in Fig. 5.

The maximum weight loss analysis results of the polymeric scaffolds present similar results with 89.3 ± 0.2% for CHL and 90.3 ± 0.8% for CHH.

The composite samples show that the progressive increase in ceramic content in the samples lead to decreased degradability of the membrane. This behaviour was expected since the Lysosome only degrades the polymer and the ceramic remains unaltered. (Khan et al. 2007; Thein-Han and Misra 2008).

Moreover, the 75%MHAp scaffolds had the lowest weight loss in both polymers compared to the MBG and MC composite. However, when compared to the other 75% ceramic content, there is no significant variation among the samples.

### Bioactivity

The in vitro bioactivity study allows for a simulation of the expected in vivo bone regeneration from the apatite formation on the materials surface that occurs when they are immersed in SBF for a specified time gap, since the SBF solution has ion concentrations similar to human blood plasma (Kokubo and Takadama 2006).

**Fig. 6:**
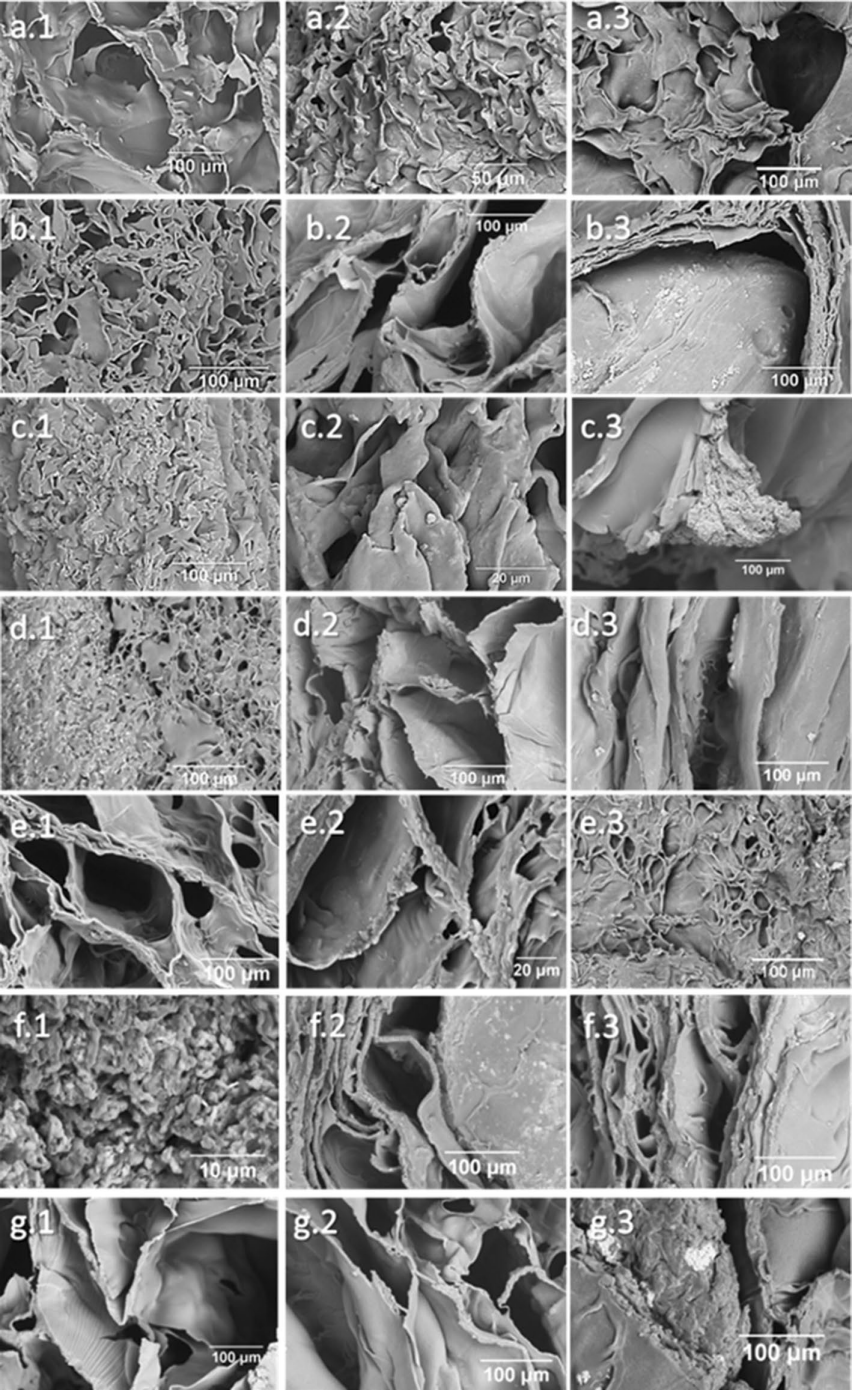
SEM images of CHL scaffolds (**a**), CHL + 25%MHAp (**b**), CHL + 25%MBG (**c**), CHL + 25%MC (**d**), CHH scaffolds (**e**), CHH + 25%MHAp (**f**) and CHH + 25%MBG (**g**) after immersion in SBF for 12 h (1), 72 h (2), and 7 days (3)

The composite scaffolds presented different responses to the test as shown in Fig. 6. Nevertheless, all the composites showed an increase of apatite precipitation with time. The precipitation begins at spots with higher rugosities or with small pores and then increases in size and distribution. The samples presented a Ca/P ratio between 1.1 and 1.75, meaning that there is an apatite and other calcium phosphates precipitation. At the end of the assay, an extensive surface coating was still not observed.

### Compression modulus

In order to analyze the compression modulus of porous composite scaffolds, the samples were tested using a mechanical testing machine. From the data obtained, the slope of the stress–strain plot at 5–10% deformation range was calculated. During the test, the pores collapsed and the structures underwent densification (Gentile et al. 2012).

**Fig. 7:**
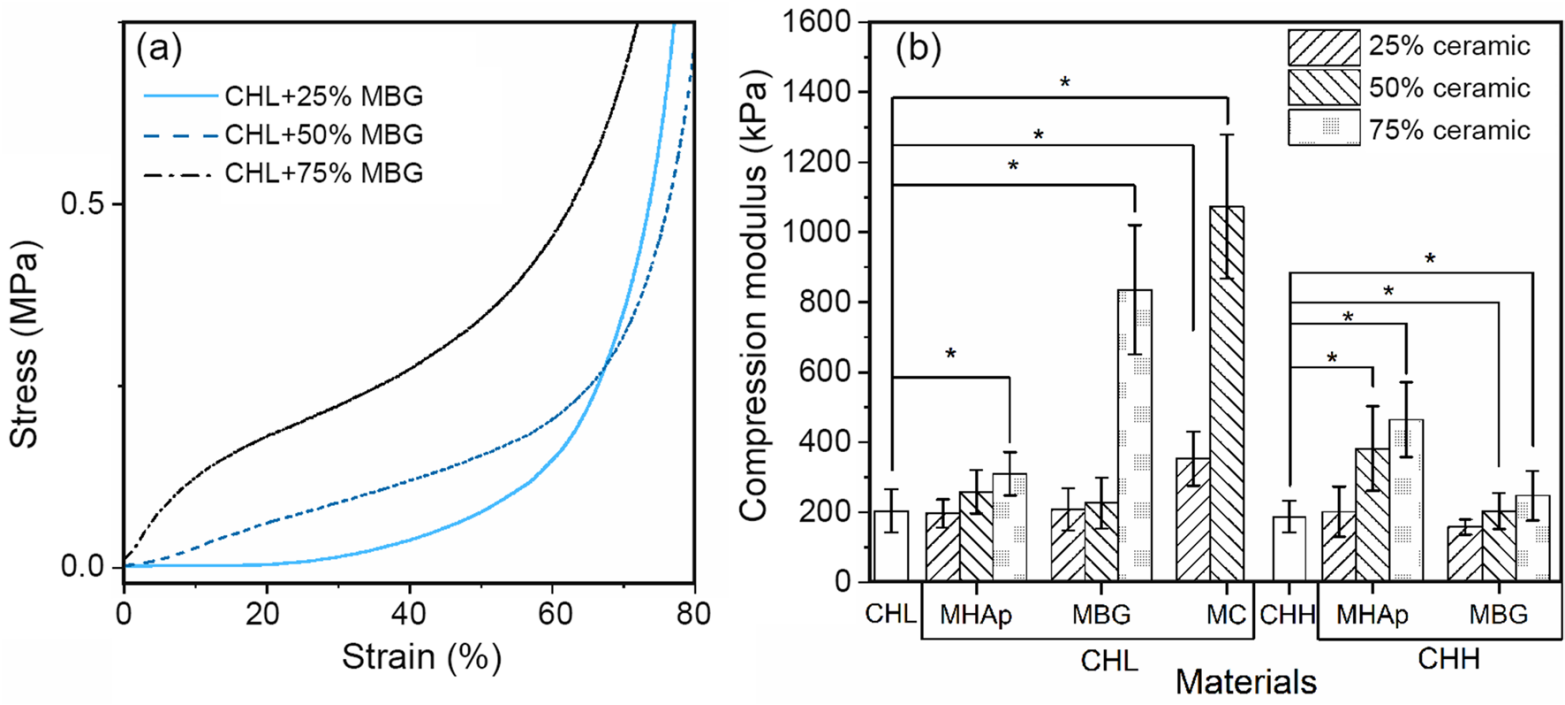
Stress–strain curves of the CHL + MBG composite scaffolds (**a**) and compression modulus of the scaffolds produced (**b**). *Significant difference between CHL and CHL composites and between CHH and CHH composites with *p* < 0.05 (*n* = 7)

With the increase in ceramic content in the scaffolds, the elastic slope tended to increase during the initial 15% of the stress–strain curve, as shown in Fig. 7a, which is due to an increase of the reinforcement effect of the ceramic filler.

The results in Fig. 7b show a similar compression modulus for the CHL and CHH scaffolds. The incorporation of ceramic materials in both low and high MW chitosan scaffolds significantly increased the compression modulus for most of the compositions tested. In the CHL scaffolds, CHL + 75% MBG scaffolds showed significant increase in compression modulus compared to CHL + 75%MHAp, which makes MBG a better mechanical reinforcement when compared to MHAp. However, in CHH scaffolds, the inverse behaviour is observed with a higher reinforcement increase for CHH + 75% MHAp scaffolds than for CHH + 75% MBG scaffolds (*p* < 0.01).

The highest value of compression modulus of all samples containing 25% ceramic was obtained for the composite produced with both mesoporous powders: CHL + 25%MC. However, the difference in compression modulus between 25% ceramic-content scaffolds was not significant. Regarding the samples containing 50% ceramic, the CHL + 50% MC scaffold has a compression modulus that significantly exceeds every other compression modulus obtained. This shows that the 1:1 mix of mesoporous ceramics is very effective in increasing the mechanical properties of the freeze-dried chitosan scaffolds.

### Cell culture studies

The cell response to the composite scaffolds was evaluated through cytotoxicity, adhesion and proliferation tests.

#### Cytotoxicity

**Fig. 8:**
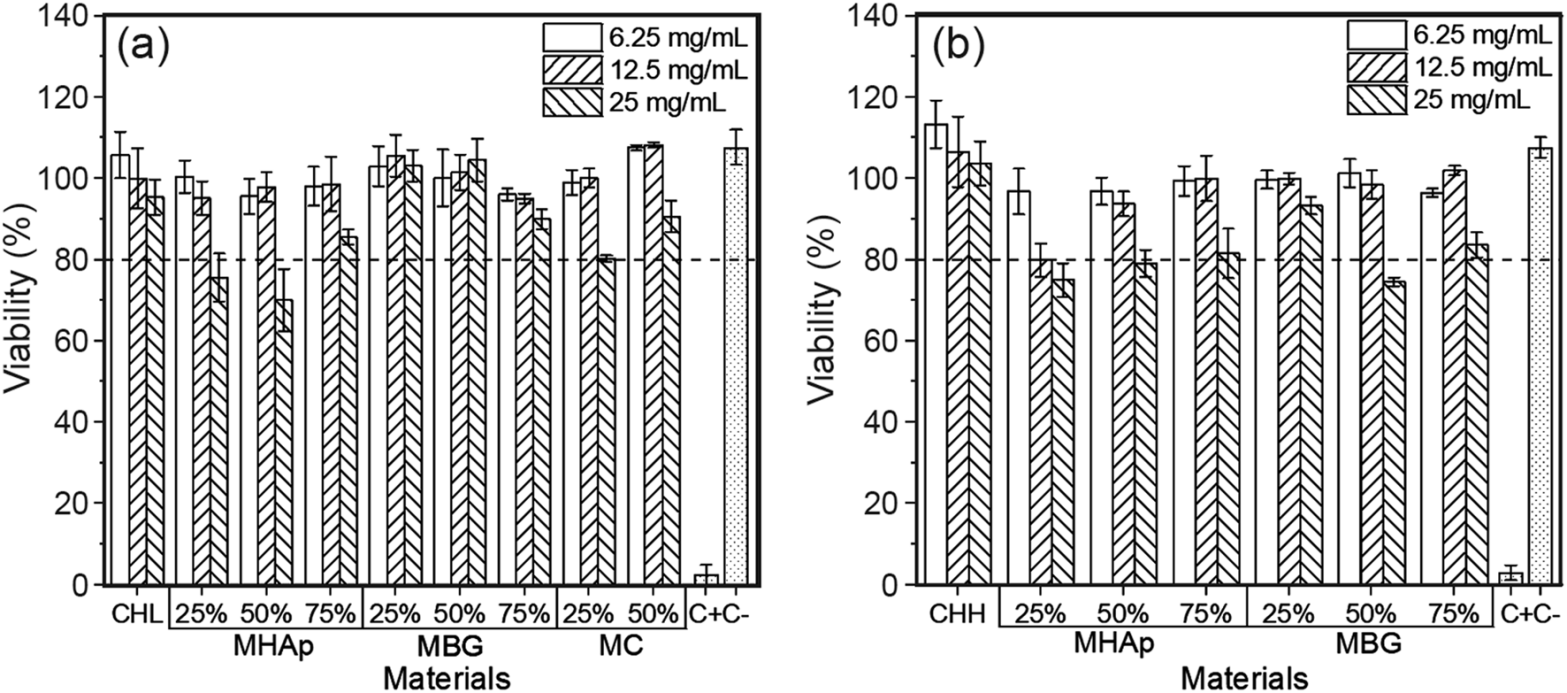
Relative cell viability in cytotoxicity tests of CH and CH composite scaffolds of CHL (**a**) and CHH (**b**)

The cytotoxicity assay of the polymeric and composite scaffolds presented in Fig. 8 shows that for all the scaffolds and for the extract concentrations of 6.25 mg/mL and 12.5 mg/mL, the relative cell viability is higher than 90%, revealing the absence of cytotoxic effects at these extract concentrations. The exception to this rule was the case of CHH + 25% MHAp scaffold that was slightly cytotoxic. Given the fact that CHH and CHH + 50% MHAp scaffolds were not cytotoxic at these extract concentrations, this exception is not worrisome. For the 25 mg/mL extract concentration, some composite scaffolds were slightly or moderately cytotoxic. Therefore, all scaffolds revealed the potential to be used in bone tissue engineering, provided the extracellular fluid in contact with both scaffolds and cells is in such amount and is renewed at a rate that prevents the concentration of lixiviates from the scaffolds to reach concentrations above those at which cytotoxic effects start to be observed.

#### Cell adhesion and proliferation

**Fig. 9:**
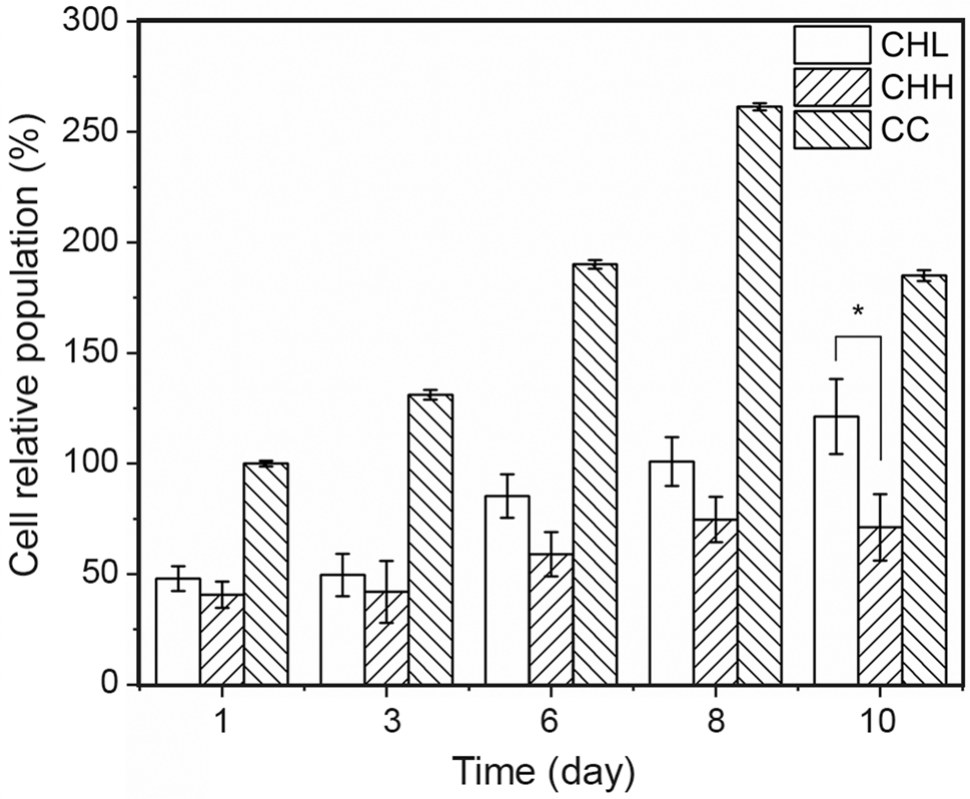
Cell relative population on CHL and CHH scaffolds. Populations are normalized to day 1 cell control (CC) values. Data obtained from a sample of six replicas for each scaffold. *Significant difference with *p* < 0.05 (*n* = 6)

One of the major purposes of this experiment is to identify the best ceramic-reinforced freeze-dried chitosan scaffold for bone regeneration. Therefore, the cell adhesion assays were performed in two stages. In the first stage, chitosan with different MWs were tested in order to identify the best cell response in what concerns adhesion and proliferation (Fig. 9), so that in the second stage all the tested composites were based on the same polymer. This selection method allowed the analysis of the second stage to focus on different ceramics under study and their concentration on the scaffold. Therefore, in the second stage, both ceramics used in composite scaffolds production were tested using their highest and lowest concentrations. This makes it possible to identify cell adhesion dependence on ceramic type and concentration.

The first-stage assay revealed that the chitosan scaffolds with CHL have a slightly higher cellular adhesion (48 ± 6%) and a significantly higher cell proliferation rate (Table 2) than the CHH scaffold (41 ± 6% cellular adhesion). As a result, in the next stage only CHL composite scaffolds are studied.

**Table 2:** Cell proliferation evaluated in CH scaffolds by the ratio between cell viability on day 10 and day 1

	CHL	CHH	CC
Proliferation rate	252 ± 46	175 ± 58	185 ± 2

Proliferation rate (PR) uncertainty is the combined standard uncertainty

In comparing the CC values, the tested materials have a slow cell proliferation with a constant population until the third day and then a steady growth throughout the experiment.

**Fig. 10:**
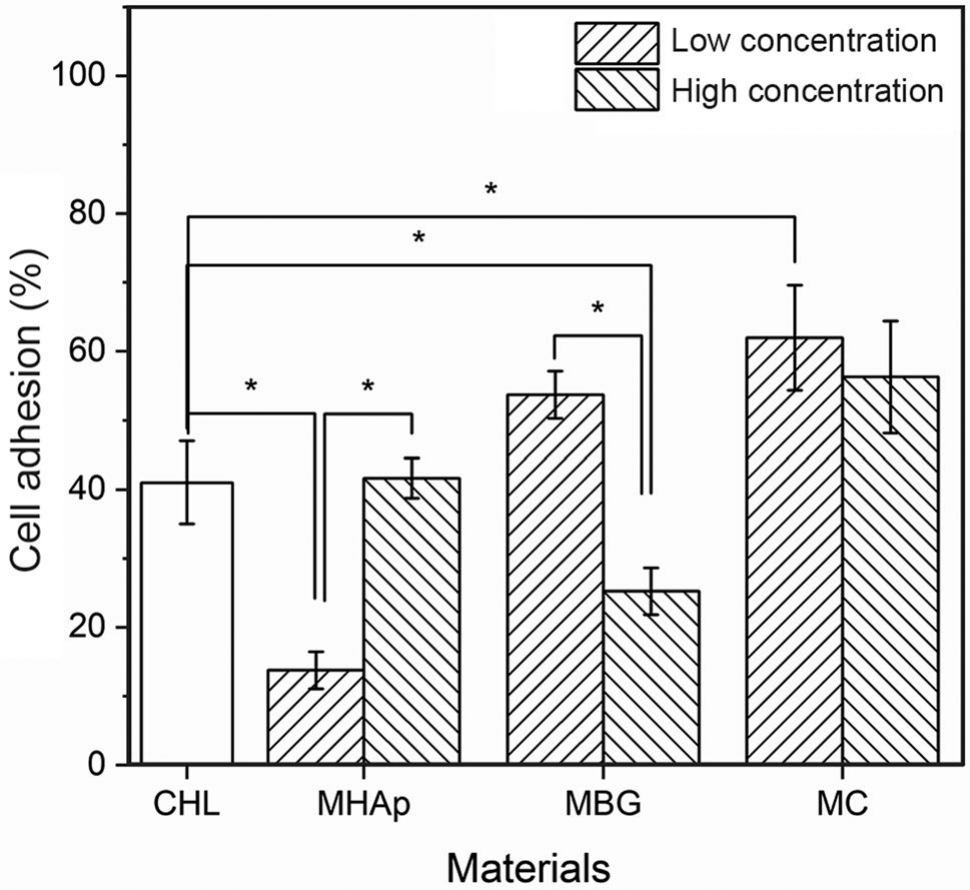
Cell adhesion to composite materials compared to polymeric scaffolds (CHL column). * Significant difference with p < 0.05 (n = 6)

The cell adhesion assay of the second stage, which evaluates cell populations 24 h after seeding, is summarized in Fig. 10. Compared to CHL scaffolds, the 25% MHAp and 75% MBG scaffolds had a reduced cell adhesion rate. The opposite is observed for the 25% MC scaffolds that had the highest nominal cell adhesion, a difference relative to the CHL scaffold statistically significant (*p* < 0.05). In MHAp samples, the cell adhesion increased with the increase of ceramic concentration, however, in the MBG scaffolds, the opposite was observed.

**Fig. 11:**
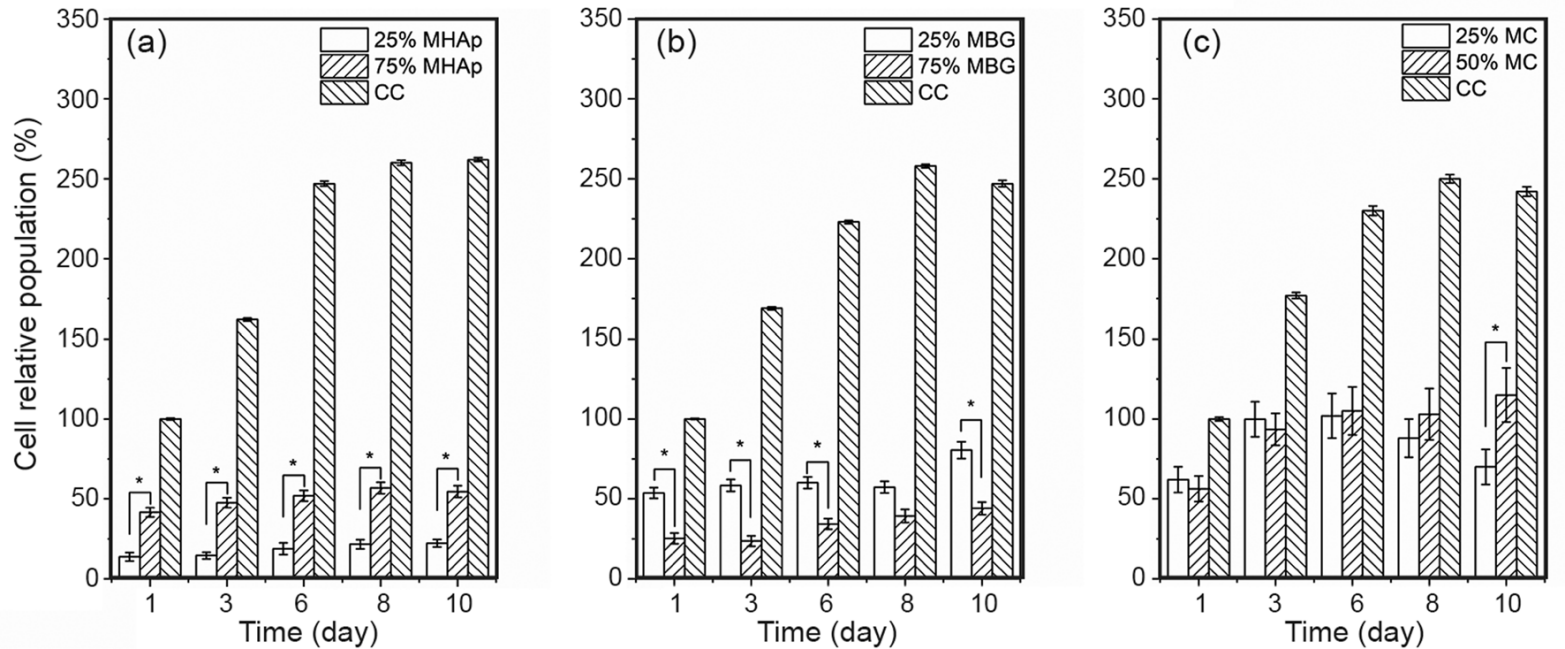
Cell relative population in composites with MHAp (**a**), MBG (**b**) and MC (**c**). *Significant difference with *p* < 0.05 (*n* = 6)

The values of the mean cell population normalized to CC values on the first day of culture are presented in Fig. 11. Cell proliferation, calculated as the ratio between cell population on day 10 and on day 1, are shown in Table 3.

**Table 3:** Cell proliferation evaluated for composite scaffolds by the ratio between cell population on day 10 and on day 1

Composite scaffold		Proliferation ratio
MHAp	25%	161 ± 36
	75%	132 ± 27
MBG	25%	125 ± 11
	75%	174 ± 18
MC	25%	131 ± 28
	50%	205 ± 39

Uncertainty is the combined standard uncertainty

Populations on the MHAp and MBG scaffolds show modest increases in number, without reaching the cell seeding density even after 10 days in culture. The 50% MC scaffolds presented the best cell proliferation ratio of all the tested samples.

**Fig. 12:**
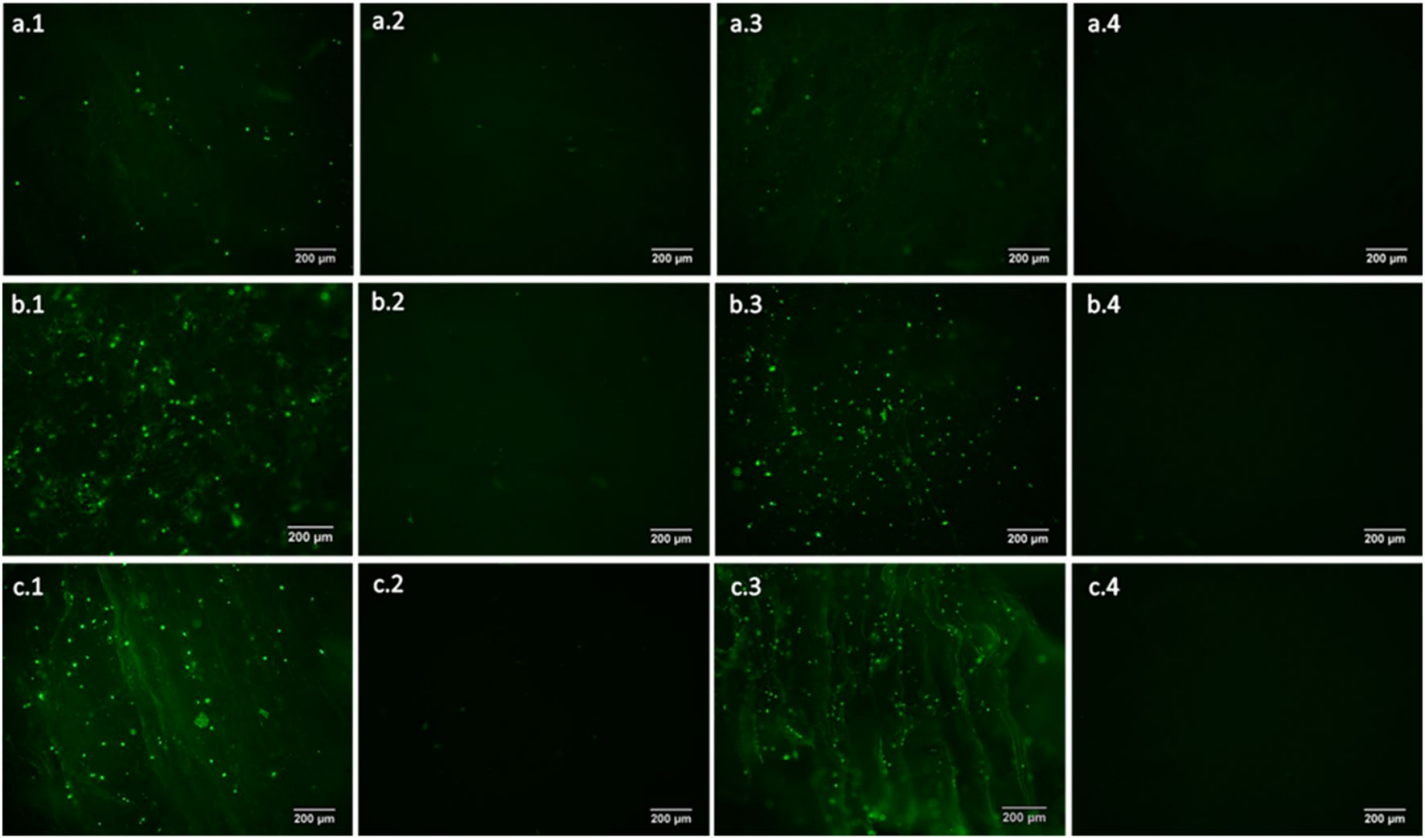
Fluorescence images of MHAp (**a**), MBG (**b**) and MC scaffolds (**c**) with low (25%) (1 and 2) and high (75% for MHAp and MBG, and 50% for MC) (3 and 4) ceramic concentrations, and their respective no cell controls (2 and 4)

The fluorescence analysis in Fig. 12 confirms the higher cell population in the MBG and MC scaffolds since the MHAp scaffolds presents a small population of stained cells. However, it is also important to mention the slight autofluorescence of both MBG and MHAp shown in (b.2) and (a.2), respectively. The MC scaffolds, with both MBG and MHAp in its composition, do not present any autofluorescence, therefore, all the spots observed in Fig. 12c ought to be due to living cells.

## Discussion

The FTIR spectra of the composites show the presence of all the materials used in their fabrication, even for the smallest ceramic concentrations, as was observed in other reports (Thein-Han and Misra 2008; Peter et al. 2010a). In the XRD diffractograms, it is only possible to identify MHAp in the composites produced due to the typical high CH signal and MBG amorphous nature (Ren et al. 2005; Thein-Han and Misra 2008; Peter et al. 2010a).

The SEM images confirm the presence of a ceramic in the scaffolds produced. A uniform distribution of the ceramics in the polymeric matrix is visible. Therefore, the majority of the ceramics appear to be well integrated within the chitosan matrices (Thein-Han and Misra 2008).

The scaffolds present a microporous structure with high porosity and wide pore size distribution that is ideal not only for cell adhesion and proliferation, but also for interlocking between the scaffolds and surrounding tissue, which will improve the mechanical stability of the implant (Loh and Choong 2013; Kang and Chang 2018; Abbasi et al. 2020). The polymeric scaffold pore organization was more alike to that obtained by Zhang et al. (2012) and Thein-Han et al. ( 2008), who used 3% (m/v) CH dissolved in acetic acid, than to that obtained by Peter et al. (Peter et al. 2010a), who used, as in this work, 2% (m/v) CH, but dissolved CH in 1% (v/v) acetic acid. This morphology difference can be due to the use of lactic acid instead of acetic acid.

In some regions of the composite scaffolds, the pores show a preferential orientation that is a consequence of the cold front propagation direction during the freezing stage of the lyophilization process (Kang et al. 1999; Deville et al. 2006; Grenier et al. 2019)and leads to very different morphologies as shown previously by Madihally et al. (1999). The samples also showed some ceramic aggregation, very common in these structures (Li et al. 2010).

The materials and morphology of the scaffolds resulted in three-stage swelling behaviour for the CH scaffolds and two stages for the composite samples. The first stage corresponds to a quick water absorption attributed to the interaction between water molecules and the chitosan hydrophilic groups (OH and NH_2_) (Pighinelli and Kucharska 2014). During the second stage, the swelling rate gradually slows down, due to the hydrogen bonds within the CH matrix, which constrains the scaffolds swelling behaviour. In the last stage, the swelling reaches the plateau due to stabilization of scaffolds (Chen et al. 2015).

All samples were able to absorb water, in proportions corresponding to several times their own weight. The individual values obtained are lower than other swelling capacities reported, which surpass 1000% (Thein-Han and Misra 2008; Peter et al. 2010a), however, the other works used a higher CH concentration (Thein-Han and Misra 2008) or other solvent (1% (v/v) acetic acid (Thein-Han and Misra 2008; Peter et al. 2010a)) that results in smaller pores with higher surface area that can increase water absorption and retention. Swelling capacity is an important property since it can lead to an increase of pore size and volume that facilitates cell infiltration and the supply of nutrients and oxygen to the interior of the composite scaffolds but can also lead to loss of mechanical properties (Peter et al. 2010b; Gentile et al. 2012; Gaihre and Jayasuriya 2018).

The swelling capacity increased with CH molecular weight. This is in contrast with the work of Thein-Han et al. (2008) who observed no significant difference. This may be explained by differences /in MW and DD: while Thein-Han et al. used 250 kDa and 400 kDa of different DD (75% and 83%, respectively), still in the present work, we used CH samples of the same DD that differ in mass by a factor of 5 (100 kDa and 500 kDa).

The MC composites show a swelling behaviour that contrasts in comparison with the CHL scaffold: while swelling decreased by 25% MC composite, it increased for the 50% MC. This behaviour may be due to water retention inside the pores of BG mesoporous morphology, since several previous works established that BG decreases scaffolds swelling capacity (Peter et al. 2009, 2010a,b; Gentile et al. 2012).

The biodegradation test has shown no significant variation between the samples and the obtained values are similar to other reported data, where the weight loss is between 5 and 15%, for 14 day degradation time (Thein-Han and Misra 2008; Han et al. 2012; Lončarević et al. 2017b). The low degradation rate proves that all the prepared scaffolds are stable for long-term performance.

The bioactivity of the scaffolds is a crucial factor for the long-term performance of tissue-engineered cell–biomaterial constructs, since the increase in scaffold bioactivity can in turn lead to improved bone cell ingrowth (osteoconduction), stable anchoring of scaffolds to host bone tissue (osseointegration), stimulation of immature host cells to develop into osteogenic cells (osteoinduction) and increased vascularization. (Rodríguez-Vázquez et al. 2015; Lončarević et al. 2017a; Turnbull et al. 2018).

The bioactivity test presented slight surface modifications in all compositions and higher apatite precipitation on the exposed surfaces for the MBG scaffolds. These results confirm the superior bioactive nature of MBG compared to MHAp (Baino et al. 2017; Ebrahimi and Sipaut 2021).

The developing of load-bearing scaffolds with high porosity is another of the major purposes of bone tissue engineering. However, the highly porous structure is obtained at the expense of mechanical strength (Ma and Choi 2001; Atkinson et al. 2021). In this trade-off, the highly porous structure is preferred in tissue engineering applications. The composite scaffolds produced have a better mechanical response (higher compression modulus) than both polymeric scaffolds while maintaining a similar porosity. The composites with higher compression modulus are those of 50% MC and 75% MBG. The 50% MC has presented values higher than other composites at 50% ceramic concentration and even 75%MHAp. This result is that obtained by Ebrahimi et al. with HAp70/BG30 but not with the results obtained by HAp50/BG50 (Ebrahimi and Sipaut 2021). The difference in results can be due to the mesoporous structure used in the present work instead of the nanosized ceramics. Furthermore, the compression modulus achieved resembles more to a porous cement (Ebrahimi and Sipaut 2021) than that of a lyophilized polymeric scaffold (Thein-Han and Misra 2008).

The evaluation of scaffolds’ cytotoxicity was performed to confirm their in vitro biocompatibility as others have previously shown (Thein-Han and Misra 2008; Peter et al. 2010a; Zhang et al. 2012).

The cell adhesion results show an increase of cell adhesion with the increase of ceramic concentration as expected for MHAp (Thein-Han and Misra 2008; Zhang et al. 2012) and the opposite behaviour for the MBG scaffolds. The 25% MBG and 75%MHAp cell adhesion results are similar to the values obtained using the polymeric scaffold. This fact could be due to morphology variations throughout the samples (Madihally and Matthew 1999; Li et al. 2010) or the slight cytotoxicity of the MBG75% scaffolds (Fig. 7) that ultimately leads to the reduction in cell adhesion and proliferation, as was shown by Luna et al. (Luna et al. 2011). The MC composite scaffolds presented the higher cell adhesion with no significant difference between the different ceramic concentrations (25% and 50% MC). Nevertheless, the 25% MC was the only sample that presented a significant increase compared to the CHL scaffold. This increase shows that using both ceramics in the composite scaffold results in a stronger structure with capacity to provide a stable surface for cell adhesion.

Comparing the proliferation rates, all samples had approximately the same PR with the exception of 50%MC that presented a much higher PR than the other produced samples, with the exception of 75%MBG. This enhanced PR may be due to the improved mechanical properties that allow a stable platform for cell adhesion and proliferation (Thein-Han and Misra 2008). Similarly, other works presented an increase in proliferation with the introduction of BG in polymeric scaffolds such as those of Kandelousi et al. (2019), Dorj et al. (2012) and Peter et al. ( 2010b).

Fluorescence microscopy of the cell cultures confirmed that human osteoblasts were able to attach, proliferate and inhabit all the tested composite scaffolds for 10 days. The MBG and MC present the greatest abundance of cells. The MBG control also presents some background fluorescence due to MBG autofluorescence, already reported by Richter et al. (2022). Cell proliferation in MBG scaffolds is not as high as it appears at first sight in fluorescence microscopy and shows similar results to MHAp, as can be seen in MHAp and MBG high concentrations that have 44 ± 4% and 54 ± 4%, respectively (Fig. 10). This test also indicates that the scaffolds support cell viability and could be a suitable support for bone regeneration applications (Thein-Han and Misra 2008; Peter et al. 2010a; Zhang et al. 2012). The cell populations do not present any preferential organization and appear to have infiltrated within the scaffold, yielding a uniform population throughout the scaffold as expected from Thein-Han et al. (2008) work. This distribution can result in faster and better tissue regeneration.

## Conclusion

Chitosan-ceramic composite scaffolds, with both MHAp and MBG, were successfully produced by lyophilization, followed by neutralization and dialysis. The scaffolds obtained present structures with interconnected pores and good ceramics distribution. From the tested polymeric scaffolds, the CHL presented better bioactivity, cell adhesion (48 ± 6%) and proliferation (252 ± 46%).

The best overall performances between the composites were the CHL + 75% MBG and CHL + 50% MC, due to their increased compression modulus (1000 kPa) and enhanced cell proliferation (174 ± 18% and 205 ± 39%, respectively). This study shows that the incorporation of several mesoporous ceramics in chitosan composite scaffolds improves their properties and can lead to better bone regeneration outcome.

## Data Availability

Data may be obtained from authors upon reasonable request.
